# Beyond COVID-19: the impact of recent pandemics on medical students and their education: a scoping review

**DOI:** 10.1080/10872981.2022.2139657

**Published:** 2022-11-04

**Authors:** Moneb S. Bughrara, Stephanie M. Swanberg, Victoria C. Lucia, Keaton Schmitz, Dawn Jung, Tracy Wunderlich-Barillas

**Affiliations:** a Department of Foundational Medical Studies, Oakland University William Beaumont School of Medicine, Rochester, MI, USA; b Moustakas Johnson Library, Michigan School of Psychology, Farmington Hills, MI, USA; c Department of Emergency Medicine, Oakland University William Beaumont School of Medicine Beaumont Health, Royal Oak, MI, USA

**Keywords:** Medical students, pandemics, COVID-19, educational adaptations, scoping review

## Abstract

**Introduction:**

Over the past two years, coronavirus disease (COVID-19) has greatly altered medical student education as well as daily life. Medical schools across the world were disrupted and had to immediately adapt the educational experience to the online environment in order to continue the delivery of quality medical education. However, COVID-19 was not the only recent pandemic. This posed the question, were similar disruptions and adaptations also seen in recent past pandemics such as Severe Acute Respiratory Syndrome (SARS) or Middle East Respiratory Syndrome (MERS) that could have prepared medical educators for COVID-19? This scoping review investigated the educational and personal impact of recent pandemics on medical students.

**Methods:**

This review followed the PRISMA-ScR guidelines for scoping reviews. Nine databases including PubMed, ERIC, and EMBASE were systematically searched using keywords and subject headings related to medical students and SARS, H1N1, MERS, Ebola, Zika, and COVID-19. Studies were limited to research studies published between 2000 and 2020 and in English. Based on exclusion and inclusion criteria, all studies were independently screened by two reviewers first by the title/abstract and then via full text. Data were extracted from the included studies and analyzed qualitatively using thematic analysis.

**Results:**

A total of 174 studies fit the criteria. Seven major themes emerged from those studies: educational adaptations and online modifications, knowledge and attitudes of students, mental wellness of students, student involvement and use of telehealth, student vaccination, physical wellness of students, and stigma.

**Conclusion:**

This review provided insights into how medical students were affected by recent pandemics and their perceptions of pivoting to online education, mental health, and knowledge of the diseases. Additionally, this review showcases the various educational adaptations that emerged uniquely during the COVID-19 pandemic, such as telehealth services or video conferencing tools, that can be utilized in a post-pandemic environment.

## Introduction

Since the World Health Organization’s first announcement of a mysterious coronavirus-related pneumonia in Wuhan, China on 9 January 2020, medical student education worldwide has been significantly impacted [1]. The rapidly spreading disease forced medical educators to abruptly alter delivery of education and examinations [2,3]. The sudden impact further altered the role of undergraduate medical students; with reports of final year medical students being allowed to graduate early in order to help with overloaded health systems [4–6]. Clinical experiences for undergraduate medical students were also drastically impacted and students were temporarily removed from clerkship environments and away rotations which led to worry about residency placement and clinical performances [7,8]. The rapid change in delivery of clinical experiences led to mass confusion, with deferring clinical rotations, involving students in telehealth services, and modifying the academic calendar becoming commonplace [7]. Such worries and disruptions, however, may not have been entirely unique to the coronavirus disease (COVID-19) pandemic.

In the last 20 years, several other worldwide pandemics and epidemics of concern have come and gone including Severe Acute Respiratory Syndrome (SARS, 2002–2004), H1N1/Swine Flu (2009–2010), Middle Eastern Respiratory Syndrome (MERS, 2012 – present), Ebola (2013–2016), and Zika (2015–2016). Each impacted daily life and education in different regions of the world [9–11]. For example, in 2003, medical education in Hong Kong and Toronto was abruptly interrupted by SARS. In Hong Kong, in-person classes were halted and lectures were provided by the means of PowerPoint slides with recorded audio [11]. Clinical sites were shut down for an extended period of time. Nonetheless, medical students still found a way to get involved during this time in Hong Kong through a public health information campaign to help raise awareness of SARS [11]. Similarly, in Toronto, clinical opportunities for students were limited at the time due to SARS [12]. These two examples highlight the impact past pandemics have also had on medical students themselves. Exploring connections across various pandemics such as SARS and COVID-19 can provide valuable information regarding innovative response and educational approaches in future health crises.

Past studies have examined the effect pandemics have had on one country, or have analyzed the effect of a singular pandemic; however, past literature has not explored the common themes and results of multiple pandemics on medical education as well as medical students across the world. Through conducting a scoping review, we may be able to better prepare our medical education system for future pandemics as well as further understand the impact such pandemics have had on medical students themselves. Furthermore, this study may highlight innovative changes and techniques to medical education that were brought upon due to extraordinary circumstances that may prove useful in a post-pandemic environment.

## Materials and methods

This scoping review followed the Preferred Reporting Items for Systematic Reviews and Meta-Analyses extension for Scoping Reviews (PRISMA-ScR) Checklist for conducting and reporting [13]. A review protocol is not available for this study.

### Eligibility criteria

Based on the review question, several eligibility criteria were developed. Studies must have been related to medical students and a worldwide pandemic or epidemic listed by the World Health Organization as a priority disease originating since 2000: SARS, H1N1/Swine Flu, MERS, Ebola, Zika, and COVID-19 [14]. Studies were limited to those in English language, published since the year 2000, and designed as research studies. Studies that focused on medical students with other health-care professionals were also included if data was distinctly reported for medical students. Exclusion criteria included non-English language studies and those published prior to 2000. Studies looking at the impact of pandemics on medical school admissions or residency applications were also excluded as we were only interested in the impact on educational programs and curriculum, not the admission process. Non-research studies including opinions, editorials, letters, commentaries, narrative reviews, and descriptive studies were also excluded.

### Systematic searches

Systematic searches were developed and conducted by a librarian in nine databases inclusive of grey literature: PubMed/MEDLINE, Cochrane Library, Embase, ERIC, Google Scholar (first 100 results), Northern Lights Life Sciences Conference Abstracts, ProQuest Dissertations and Theses, Scopus, and Web of Science. Search terms included a combination of keywords and subject headings related to medical students (‘Students, Medical’[Mesh], medical student, medical education, medical school, etc.) and pandemics (‘severe acute respiratory syndrome’, SARS, ‘Middle Eastern Respiratory Syndrome’, MERS, Ebola, H1N1, influenza A, coronavirus, ‘Coronavirus Infections’[Mesh], COVID, ‘Disease Outbreaks’[Mesh], pandemic, epidemic, etc.). A complete list of search strategies is included in Appendix A. Searches were initially conducted on 10 June 2020 and rerun on 1 December 2020 to capture the emerging COVID-19 literature.

### Screening process

All citations retrieved from the searches were uploaded into the Covidence systematic review management software (https://www.covidence.org/) for screening and duplicates were removed by the software. A pilot screening of 20 randomly selected citations was then conducted to establish inter-rater reliability among team members. Two team members then independently screened the title/abstract of each citation against the inclusion/exclusion criteria. Any differences in interpretation were resolved by a third reviewer. Following title/abstract screening, full-text screening of the remaining studies was similarly performed by two independent reviewers with any disagreements resolved by a third reviewer.

### Data extraction and synthesis

Following the screening process, the team developed a data extraction form of 15 elements to chart data from the included studies. Data categories included publication information, study purpose, methods, and results (see Appendix B for data extraction form). The form was then converted into a Google spreadsheet for recording and piloted by all team members using three of the included studies. Primary and secondary reviewers were then assigned to extract data from each included study. The primary reviewer completed the data extraction for each study and then the secondary reviewer reviewed and noted any discrepancies. A third reviewer then resolved and combined the data into a single entry. Critical appraisal of included studies was not conducted as part of this study.

In synthesizing the qualitative data obtained from the data extraction, the team used thematic analysis through inductive coding. In inductive coding, themes are generated by directly reviewing the data to generate a set of themes [15]. As such, the team met to collectively review and inductively code all included studies and identified several common themes for organizing and reporting. Then, smaller teams of 2–3 were formed for each theme. Sub-themes were also identified through inductive coding and then narratively summarized by each team. A summary of themes and sub-themes is reported in the following section.

## Results

### Study characteristics

**Figure 1.**
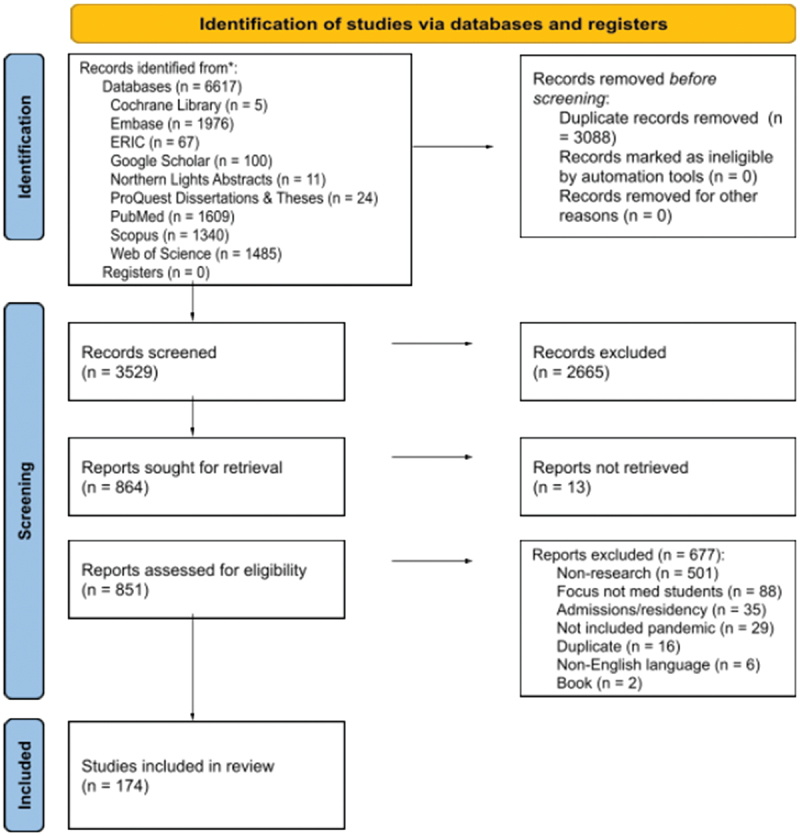
Flow diagram of screening process and included studies.

**Figure 2.**
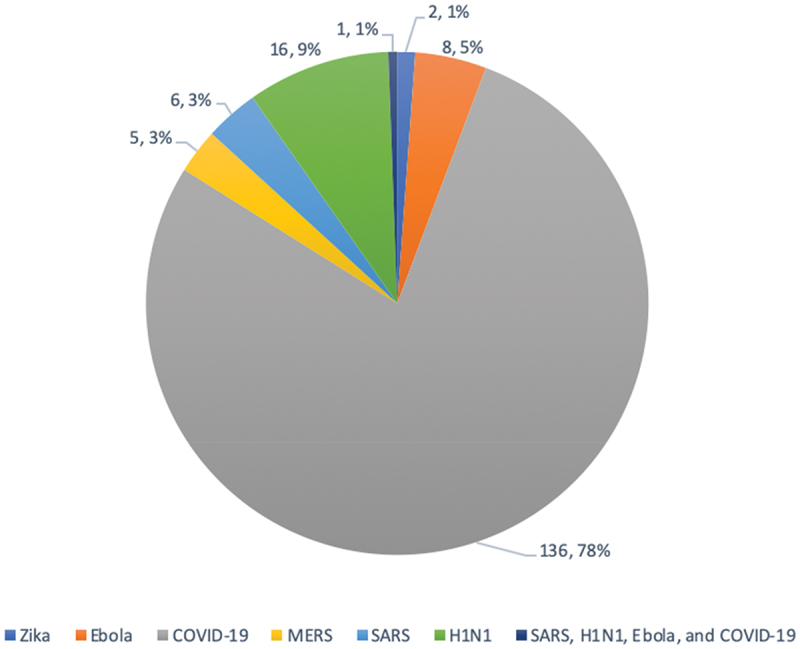
Distribution of articles for each pandemic (n, %) (n = 174).

**Figure 3.**
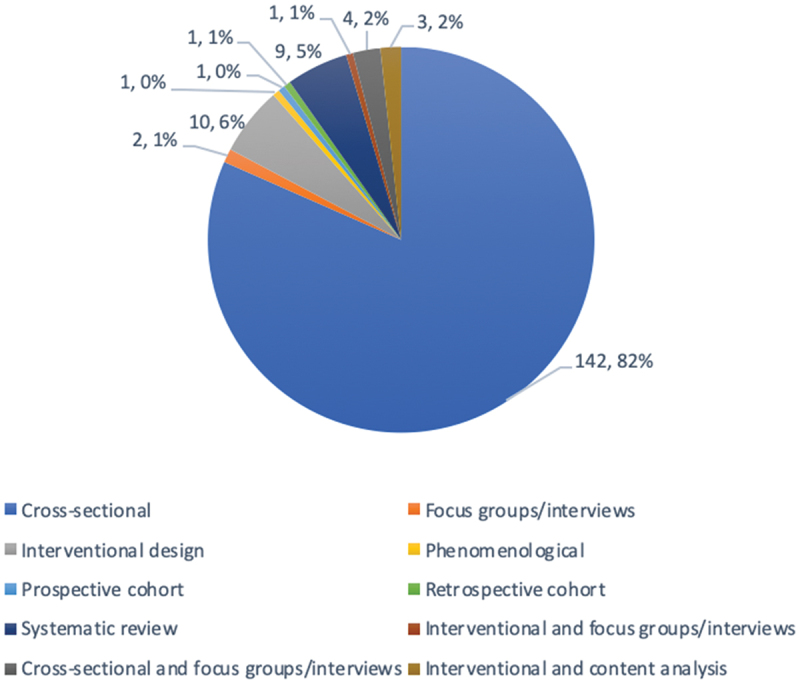
Distribution of articles for each study type (n,%) (n = 174).

Through database searching, 3,555 records were identified in June 2020 and an additional 3,062 when searches were run a second time in December 2020 to capture the emerging COVID-19 literature. With removal of duplicates, 3,529 total records were screened for this study (Figure 1). Following screening and data extraction, a total of 174 studies met the inclusion criteria and are summarized in Table 1. Thirty-four studies were published prior to 2020 with 140 published in 2020. The majority were related to COVID-19 (n = 136), with representation from H1N1 (n = 16), Ebola (n = 8), SARS (n = 6), MERS (n = 5), Zika (n = 2), and one covering multiple pandemics (n = 1) (Figure 2). A total of 42 countries were represented with the United States (n = 32), India (n = 19), Saudi Arabia (n = 14), China (n = 12), and the United Kingdom (n = 10) being the five most represented. Study methods included quantitative (n = 151), qualitative (n = 15), and mixed methods (n = 8). Study designs included cross-sectional studies (n = 146), interventional design (n = 14), systematic reviews (n = 9), focus groups/interviews (n = 7), content analysis (n = 3) as well as a phenomenological study, prospective cohort study, and retrospective cohort study (n = 1 each) (Figure 3).

**Table 1.** Information regarding included studies.

Ref#	Author (Last Name, First Initial)	Year	Country	Theme(s)	Study methods	Study design	Populations studied	Number of medical student participants	COVID-19, SARS, MERS, Zika, Ebola, H1N1/Swine Flu, or general pandemic
142	Abbas, M	2020	Canada	Mental Wellness	Quantitative	Cross-sectional	Medical Students	627	COVID-19
16	Abbasi, S	2020	Pakistan	Educational Adaptations and Online Modifications	Quantitative	Cross-sectional	Medical Students, Dental Students	204	COVID-19
17	Abdulghani, H	2020	Saudi Arabia	Mental Wellness + Educational Adaptations and Online Modifications	Quantitative	Cross-sectional	Medical Students	243	COVID-19
18	Abraham, H	2020	USA	Educational Adaptations and Online Modifications + Student Involvement and Telehealth	Mixed methods	Interventional Design; Focus Group	Medical Students	20	COVID-19
19	Adams, C	2020	USA	Educational Adaptations and Online Modifications	Quantitative	Cross-sectional	Medical Students, Transitional Year Interns	35	COVID-19
84	Aker S	2020	Turkey	Student Involvement and Telehealth + Student Knowledge and Attitudes	Quantitative	Cross-sectional	Medical Students	1375	COVID-19
20	Al-Balas, M	2020	Jordan	Educational Adaptations and Online Modifications	Quantitative	Cross-sectional	Medical Students	652	COVID-19
87	Al-Mohrej, A	2017	Saudi Arabia	Student Knowledge and Attitudes	Quantitative	Cross-sectional	Medical Students	136	MERS
88	Al-Rabiaah, A	2020	Saudi Arabia	Student Knowledge and Attitudes	Quantitative	Cross-sectional	Medical Students	174	MERS
85	Alao, M	2020	Nigeria	Student Knowledge and Attitudes	Quantitative	Cross-sectional	Medical Students, Nurses, Lab Scientists, Opticians, Residents, Physicians, Physiotherapists	72	COVID-19
86	Alaydrus, L	2019	Malaysia	Student Knowledge and Attitudes	Quantitative	Cross-sectional	Medical Students, Dental Students, Pharmacy Students, Physiotherapy Students, Nursing Students	50	Ebola
21	Alkhowailed, M	2020	Saudi Arabia	Educational Adaptations and Online Modifications	Quantitative	Cross-sectional	Medical Students	674	COVID-19
22	Alpert, J	2020	USA	Educational Adaptations and Online Modifications	Quantitative	Cross-sectional	Medical Students	68	COVID-19
167	AlSaif, H	2020	Saudi Arabia	Student Involvement and Telehealth	Quantitative	Cross-sectional	Medical Students	134	COVID-19
23	Alsoufi, A	2020	Libya	Educational Adaptations and Online Modifications	Quantitative	Cross-sectional	Medical Students	3348	COVID-19
89	Alzoubi, H	2020	Jordan	Student Knowledge and Attitudes	Quantitative	Cross-sectional	Medical Students, Nonmedical students	323	COVID-19
24	Anderi, E	2020	USA	Educational Adaptations and Online Modifications	Quantitative	Cross-sectional	Medical Students	55	COVID-19
90	Asaad, A	2019	Saudi Arabia	Student Knowledge and Attitudes	Quantitative	Cross-sectional	Health College Students: Medicine, Dentistry, Pharmacy, Physiotherapy, Radiology, Medical Laboratory.	118	MERS
91	Ashcroft, J	2020	Multicountry	Student Knowledge and Attitudes	Qualitative	Systematic Review	Medical Students	Not applicable	COVID-19
168	Astorp, M	2020	Denmark	Student Involvement and Telehealth	Quantitative	Cross-sectional	Medical Students	486	COVID-19
169	Bickerton, L	2020	USA	Student Involvement and Telehealth	Quantitative	Cross-sectional	Medical Students	29	COVID-19
143	Bolatov, A	2020	Kazakhstan	Mental Wellness	Quantitative	Cross-sectional	Medical Students	1417	COVID-19
92	Bonilla-Asalde, C	2020	Peru	Student Knowledge and Attitudes	Quantitative	Cross-sectional	Health Science Students: Medical, Nursing, Dentistry, Medical Technology, Obstetrics, Nutrition	653	COVID-19
177	Brandt, C	2011	Germany	Student Vaccination	Quantitative	Cross-sectional	Medical Students, Physicians, Nurses, Medical Technicians, Administrative Personnel, Maintenance, Catering, Workshop, Transport, Others	322	H1N1
93	Brorsson, A	2002	Sweden	Student Knowledge and Attitudes	Quantitative	Cross-sectional	Medical Students	521	Ebola
94	Byrnes, Y	2020	USA	Student Knowledge and Attitudes	Quantitative	Cross-sectional	Medical Students	1668	COVID-19
95	Caliskan, F	2020	Turkey	Student Knowledge and Attitudes + Student Involvement and Telehealth	Quantitative	Cross-sectional	Medical Students	860	COVID-19
25	Camargo	2020	Multicountry	Educational Adaptations and Online Modifications	Qualitative	Systematic Review	Medical Students	Not applicable	COVID-19
26	Carrascosa, M	2020	Brazil	Student Knowledge and Attitudes + Educational Adaptations and Online Modifications	Quantitative	Cross-sectional	Medical Students	317	COVID-19
170	Carson, S	2020	USA	Student Involvement and Telehealth	Quantitative	Cross-sectional	Medical Students	17	COVID-19
96	Caves, N	2005	Hong Kong	Student Knowledge and Attitudes	Quantitative	Cross-sectional	Medical Students	35	SARS
27	Chandrasinghe, P	2020	Sri Lanka	Educational Adaptations and Online Modifications	Quantitative	Interventional Design	Medical Students	1047	COVID-19
28	Choi, B	2020	United Kingdom	Educational Adaptations and Online Modifications	Quantitative	Cross-sectional	Medical Students	440	COVID-19
29	Co	2020	China	Educational Adaptations and Online Modifications	Quantitative	Cross-sectional	Medical Students	30	COVID-19
30	Coffey, C	2020	USA	Educational Adaptations and Online Modifications	Quantitative	Cross-sectional	Medical Students	96	COVID-19
31	Compton, S	2020	Republic of Singapore	Educational Adaptations and Online Modifications	Quantitative	Cross-sectional	Medical Students	179	COVID-19
144	Coyle, C	2020	United Kingdom	Mental Wellness	Quantitative	Cross-sectional	Medical students, Residents	1909	COVID-19
32	Cuschieri, S	2020	Republic of Malta	Educational Adaptations and Online Modifications	Quantitative	Cross-sectional	Medical Students	172	COVID-19
33	Darnton, R	2020	United Kingdom	Educational Adaptations and Online Modifications + Student Involvement and Telehealth	Qualitative	Interviews	Medical Students, Physicians	13	COVID-19
97	Datta, R	2020	India	Student Knowledge and Attitudes	Quantitative	Cross-sectional	Medical Students; Physicians; medical practitioners who were nonspecialists	255	COVID-19
178	de Souza, E	2012	Brazil	Student Vaccination	Quantitative	Cross-sectional	Medical Students	678	H1N1
34	Dedeilia, A	2020	Multicountry	Educational Adaptations and Online Modifications	Qualitative	Systematic Review	Medical Students, Residents, Fellows	Not applicable	COVID-19
35	Deepika, V	2020	India	Educational Adaptations and Online Modifications	Qualitative	Systematic Review	Medical Students	Not applicable	COVID-19
36	DePietro, D	2020	USA	Educational Adaptations and Online Modifications	Quantitative	Interventional Design	Medical Students	10	COVID-19
37	Deponti	2020	Italy	Educational Adaptations and Online Modifications	Quantitative	Interventional Design	Medical Students	115	COVID-19
38	Desai, D	2020	India	Educational Adaptations and Online Modifications	Quantitative	Cross-sectional	Undergraduates, Postgraduates, Faculty	165	COVID-19
145	Dhahri, A	2020	Pakistan	Mental Wellness	Quantitative	Cross-sectional	Medical Students, Dental Students	2263	COVID-19
39	Dost, S	2020	United Kingdom	Educational Adaptations and Online Modifications	Quantitative	Cross-sectional	Undergraduate Medical Students, Graduate Medical Students	2595	COVID-19
40	Dow, N	2020	United Kingdom	Educational Adaptations and Online Modifications	Quantitative	Interventional Design	Medical Students	162	COVID-19
171	Drexler, R	2020	Germany	Student Involvement and Telehealth	Quantitative	Cross-sectional	Medical Students	137	COVID-19
185	Duong, T	2020	Vietnam	Physical Wellness	Quantitative	Cross-sectional	Undergraduate Medical Students; Undergraduate Nursing Students	5765	COVID-19
41	Durfee, S	2020	USA	Educational Adaptations and Online Modifications	Quantitative	Cross-sectional	Medical Students	56	COVID-19
146	Dwivedi, D	2020	India	Mental Wellness	Quantitative	Cross-sectional	Medical Students	924	COVID-19
98	Echoru, I	2020	Uganda	Student Knowledge and Attitudes	Quantitative	Cross-sectional	Undergraduate Medical Students, Faculty	52	COVID-19
100	El-Masry, E	2020	Saudi Arabia	Student Knowledge and Attitudes	Quantitative	Cross-sectional	Medical Students	196	MERS
99	Elhadi, M	2020	Libya	Student Knowledge and Attitudes	Quantitative	Cross-sectional	Medical Students, Dentistry Students, Pharmacy Students, Nursing Students, Medical Technology Students, Veterinary Science Students	2547	COVID-19
141	Elhadi, M	2020	Libya	Mental Wellness	Quantitative	Cross-sectional	Medical Students	2430	COVID-19
101	Elrggal, M	2018	Saudi Arabia	Student Knowledge and Attitudes	Quantitative	Cross-sectional	Medical Students, Dental, Students, Pharmacy Students, Nursing Students	80	MERS
42	Elsalem, L	2020	Jordan	Educational Adaptations and Online Modifications + Mental Wellness	Quantitative	Cross-sectional	Medical Students, Dental, Students, Pharmacy Students, Nursing Students, Applied Medical Sciences Students	523	COVID-19
43	Escalon, M	2020	USA	Educational Adaptations and Online Modifications	Quantitative	Cross-sectional	Medical Students, Physicians, Residents, Fellows, Interns	111	COVID-19
147	Essangri, H	2020	Morocco	Mental Wellness	Quantitative	Cross-sectional	Medical Students	549	COVID-19
3	Eurboonyanun, C	2020	Thailand	Educational Adaptations and Online Modifications	Quantitative	Retrospective Cohort	Medical Students	227	COVID-19
179	Faresjo, T	2012	USA	Student Vaccination	Quantitative	Cross-sectional	Medical students, Nursing Students	222	H1N1
44	Fatani, T	2020	Saudi Arabia	Educational Adaptations and Online Modifications	Quantitative	Cross-sectional	Medical Students	662	COVID-19
102	Gao, Z	2020	China	Student Knowledge and Attitudes	Quantitative	Cross-sectional	Medical Students, Non-Medical Students	388	COVID-19
45	Giordano, L	2020	Multicountry	Educational Adaptations and Online Modifications	Qualitative	Systematic Review	Medical Students, Residents	Not applicable	COVID-19
46	Gordon, M	2020	Multicountry	Educational Adaptations and Online Modifications	Qualitative	Systematic Review	Medical Students, Residents	Not applicable	COVID-19
104	Haque, A	2020	Pakistan	Student Knowledge and Attitudes	Quantitative	Cross-sectional	Medical Students, Physicians	306	COVID-19
103	Harapan, H	2019	Indonesia	Student Knowledge and Attitudes	Quantitative	Cross-sectional	Medical Students, Residents, Physicians	409	Zika
180	Hasan, F	2018	Pakistan	Student Vaccination	Quantitative	Cross-sectional	Medical Students, Dental Students	450	H1N1
105	Hisam, A	2016	Pakistan	Student Knowledge and Attitudes	Quantitative	Cross-sectional	Medical Students, Dental Students	258	Ebola
106	Hsu, L	2011	Singapore	Student Knowledge and Attitudes	Quantitative	Cross-sectional	Medical Students	314	H1N1
148	Huang, G	2004	China	Mental Wellness	Quantitative	Cross-sectional	Medical Students, Non-Medical Students	150	SARS
172	Hughes T	2020	England	Student Involvement and Telehealth	Mixed methods	Cross-sectional, Interviews	Medical Students, Physicians, Patients	33	COVID-19
107	Hussain, Z	2012	Pakistan	Student Knowledge and Attitudes	Quantitative	Cross-sectional	Medical Students	251	H1N1
47	Iqbal, M	2020	Saudi Arabia	Educational Adaptations and Online Modifications	Qualitative	Cross-sectional	Medical Students	203	COVID-19
48	Jack, M	2020	USA	Educational Adaptations and Online Modifications	Quantitative	Cross-sectional	Medical Students, Residents	6	COVID-19
173	Jackman, D	2020	Canada	Student Involvement and Telehealth	Qualitative	Phenomenological	Medical Students, Nursing Students	8	COVID-19
149	Ji, D	2017	Sierra Leone	Mental Wellness	Quantitative	Cross-sectional	Medical Students, Medical Staff, Patients	22	Ebola
108	John, A	2017	USA	Student Knowledge and Attitudes	Quantitative	Cross-sectional	Medical Students, Residents, Fellows, Physicians	27	Ebola
187	Joshi, A	2020	India	Physical Wellness	Quantitative	Cross-sectional	Medical Students	149	COVID-19
49	Khalil, R	2020	Saudi Arabia	Educational Adaptations and Online Modifications	Qualitative	Focus group	Medical Students	60	COVID-19
109	Khasawneh, A	2020	Jordan	Student Knowledge and Attitudes	Quantitative	Cross-sectional	Medical Students	1,404	COVID-19
110	Khowaja, Z	2011	Pakistan	Student Knowledge and Attitudes	Quantitative	Cross-sectional	Medical Students	396	H1N1
50	Kim, S	2020	South Korea	Educational Adaptations and Online Modifications	Quantitative	Cross-sectional	Medical Students	161	COVID-19
51	Kolcu, G.	2020	Turkey	Educational Adaptations and Online Modifications	Quantitative	Cross-sectional	Medical Students	941	COVID-19
111	Komasawa, N	2020	Japan	Student Knowledge and Attitudes	Quantitative	Cross-sectional	Medical Students	123	COVID-19
52	Krawiec, C	2020	USA	Educational Adaptations and Online Modifications	Quantitative	Cross-sectional	Medical Students	12	COVID-19
150	Kumar, A	2020	India	Mental Wellness	Quantitative	Cross-sectional	Medical Students	331	COVID-19
186	Kumar, S	2020	India	Physical Wellness	Quantitative	Cross-sectional	Medical Students	760	COVID-19
151	Lasheras, I	2020	Multicountry	Mental Wellness	Qualitative	Systematic Review	Medical Students	Not applicable	COVID-19
181	Lee, S	2012	United Kingdom	Student Vaccination	Quantitative	Cross-sectional	Medical Students	203	H1N1
53	Liang, S	2020	USA	Educational Adaptations and Online Modifications	Qualitative	Interventional Design	Medical Students	8	COVID-19
54	Lieberman, J	2020	USA	Educational Adaptations and Online Modifications	Mixed methods	Interventional Design; Content Analysis	Medical Students	14	COVID-19
152	Liu, J	2020	China	Mental Wellness	Quantitative	Cross-sectional	Medical Students	217	COVID-19
112	Loda, T	2020	Germany	Mental Wellness + Student Knowledge and Attitudes	Quantitative	Cross-sectional	Medical Students	372	COVID-19
113	Loh, L	2005	Malaysia	Student Knowledge and Attitudes	Quantitative	Cross-sectional	Medical Students	204	SARS
153	Lyons, Z	2020	Australia	Mental Wellness	Quantitative	Cross-sectional	Medical Students	297	COVID-19
114	Maheshwari, S	2020	India	Student Knowledge and Attitudes	Quantitative	Cross-sectional	Medical Students	354	COVID-19
115	Mahwish, R	2015	Pakistan	Student Knowledge and Attitudes	Quantitative	Cross-sectional	Medical Students	153	Ebola
55	Manalo, T	2020	USA	Educational Adaptations and Online Modifications	Quantitative	Interventional Design	Medical Students	9	COVID-19
174	Martin, A	2020	Multicountry	Student Involvement and Telehealth	Qualitative	Systematic Review	Medical Students	Not Applicable	SARS, H1N1, Ebola, COVID-19
56	Martinez, L	2020	USA	Educational Adaptations and Online Modifications	Quantitative	Cross-sectional	Medical Students	47	COVID-19
175	Masumbuko Claud, K	2020	Democratic Republic of Congo	Student Involvement and Telehealth	Quantitative	Cross-sectional	Medical Students, Public	355	Ebola
116	Matusiak, Ł	2020	Poland	Student Knowledge and Attitudes	Quantitative	Cross-sectional	Medical Students	1170	COVID-19
182	Mavros, M	2010	Greece	Student Vaccination	Quantitative	Cross-sectional	Medical Students	922	H1N1
117	May, L	2010	USA	Student Vaccination + Student Knowledge and Attitudes	Mixed methods	Cross-sectional, Interviews	Medical Students, Residents	194	H1N1
57	Mehta, M	2020	India	Educational Adaptations and Online Modifications	Quantitative	Cross-sectional	Medical Students	120	COVID-19
154	Meo, S	2020	Saudi Arabia	Mental Wellness	Quantitative	Cross-sectional	Medical Students	530	COVID-19
58	Michener, A	2020	USA	Educational Adaptations and Online Modifications	Quantitative	Cross-sectional	Medical Students	23	COVID-19
118	Mishra, A	2020	India	Student Knowledge and Attitudes	Quantitative	Cross-sectional	Medical Students, Residents	112	COVID-19
59	Mitra, M	2020	India	Educational Adaptations and Online Modifications	Quantitative	Cross-sectional	Medical Students, Residents	218	COVID-19
119	Modi, P	2020	India	Student Knowledge and Attitudes	Quantitative	Cross-sectional	Medical Students, Residents, Faculty (medical, dental, nursing, physical therapy), Non-Clinical Staff, Administration, Allied Health Professionals	517	COVID-19
60	Monday, L	2020	USA	Educational Adaptations and Online Modifications	Quantitative	Interventional Design	Medical Students	89	COVID-19
61	Nagji, A	2020	Canada	Educational Adaptations and Online Modifications	Mixed methods	Interventional Design; Content Analysis	Medical Students, Residents, Faculty	23	COVID-19
120	Naing, C	2011	Malaysia	Student Vaccination + Student Knowledge and Attitudes	Quantitative	Cross-sectional	Medical Students	264	H1N1
155	Nakhostin-Ansari, A	2020	Iran	Mental Wellness	Quantitative	Cross-sectional	Medical Students	323	COVID-19
62	Nepal, S	2020	Nepal	Educational Adaptations and Online Modifications	Quantitative	Cross-sectional	Medical Students	226	COVID-19
121	Neupane, H	2020	Nepal	Student Knowledge and Attitudes	Quantitative	Cross-sectional	Medical Students, Physicians, Nurses, Dentists, Allied Health Professionals	43	COVID-19
63	Newcomb, A	2020	USA	Educational Adaptations and Online Modifications	Mixed methods	Cross-sectional; Focus Group	Medical Students	5	COVID-19
122	Nguyen, D	2020	Vietnam	Student Knowledge and Attitudes	Quantitative	Cross-sectional	Medical Students	2019	COVID-19
123	Nguyen, H	2020	Vietnam	Student Knowledge and Attitudes	Quantitative	Cross-sectional	Medical Students	5423	COVID-19
156	Nihmath Nisha, N	2020	India	Mental Wellness	Quantitative	Cross-sectional	Medical Students	359	COVID-19
124	Norton, E	2020	United Kingdom	Student Knowledge and Attitudes	Quantitative	Cross-sectional	Medical Students, Residents	1909	COVID-19
125	Olaimat, A	2020	Jordan	Student Knowledge and Attitudes	Quantitative	Cross-sectional	Undergraduate and Graduate Students (human sciences, medical sciences, engineering sciences, agriculture, general sciences)	535	COVID-19
127	Ozer, A	2011	Turkey	Student Vaccination + Student Knowledge and Attitudes	Quantitative	Cross-sectional	Medical Students	68	H1N1
126	Ozer, A	2016	Turkey	Student Knowledge and Attitudes	Quantitative	Cross-sectional	Medical Students, Nursing Students, Midwifery Students	984	Ebola
64	Park, J	2020	South Korea	Student Knowledge and Attitudes + Educational Adaptations and Online Modifications	Quantitative	Cross-sectional	Medical Students	73	COVID-19
176	Patel, J	2020	United Kingdom	Student Involvement and Telehealth	Quantitative	Cross-sectional	Medical Students	132	COVID-19
183	Paula, S	2016	Brazil	Student Vaccination	Quantitative	Cross-sectional	Medical Students	144	H1N1
128	Puri, S.	2011	India	Student Knowledge and Attitudes	Quantitative	Cross-sectional	Medical Students, Residents, Nurses	155	H1N1
129	Purssell	2011	United Kingdom	Student Vaccination + Student Knowledge and Attitudes	Quantitative	Cross-sectional	Medical Students, Nursing Students, Midwifery Students, Other Health Students, Non-Health Students	67	H1N1
130	Rabbani, S	2018	United Arab Emirates	Student Knowledge and Attitudes	Quantitative	Cross-sectional	Medical Students, Dental, Students, Pharmacy Students, Nursing Students	257	Zika
65	Rafi, A	2020	India	Educational Adaptations and Online Modifications	Quantitative	Cross-sectional	Medical Students	402	COVID-19
131	Rahman, M	2020	Malaysia	Student Knowledge and Attitudes	Quantitative	Cross-sectional	Medical Students	467	COVID-19
66	Rajab, M	2020	Saudi Arabia	Educational Adaptations and Online Modifications	Quantitative	Cross-sectional	Medical Students, Public Health Students, Other Graduate Students, Faculty	139	COVID-19
157	Remitha, N	2020	Indonesia	Mental Wellness	Quantitative	Cross-sectional	Undergraduate Medical Students, Professional Doctor Students	175	COVID-19
67	Rishi, S	2020	India	Educational Adaptations and Online Modifications	Quantitative	Cross-sectional	Medical Students	1200	COVID-19
68	Robertson, B	2020	USA	Educational Adaptations and Online Modifications	Qualitative	Cross-sectional	Medical Students, Nursing Students	11	COVID-19
184	Rodas, J	2012	Hong Kong	Student Vaccination	Quantitative	Prospective Cohort	Medical Students, Non-Medical Students	56	H1N1
69	Roy, H	2020	India	Educational Adaptations and Online Modifications	Quantitative	Cross-sectional	Bachelor of Medicine, Bachelor of Surgery (MBBS) Students	182	COVID-19
188	Rzymski, P.	2020	Poland	Stigma	Quantitative	Cross-sectional	Medical Students	85	COVID-19
132	Saleem, M	2020	Saudi Arabia	Student Knowledge and Attitudes	Quantitative	Cross-sectional	Medical Students, Dental Students	180	COVID-19
70	Samueli, B	2020	Israel	Educational Adaptations and Online Modifications	Quantitative	Cross-sectional	Medical Students	25	COVID-19
71	Sandhaus, Y	2020	Israel	Educational Adaptations and Online Modifications	Mixed methods	Cross-sectional, Interviews	Medical Students	70	COVID-19
72	Sandhu, N	2020	USA	Educational Adaptations and Online Modifications	Quantitative	Cross-sectional	Medical Students, Residents, Faculty	26	COVID-19
158	Saraswathi, I.	2020	India	Mental Wellness	Quantitative	Interventional Design	Medical Students	217	COVID-19
133	Sari, I	2020	Indonesia	Student Knowledge and Attitudes	Quantitative	Cross-sectional	Medical Students	368	COVID-19
73	Shahrvini, B	2020	USA	Educational Adaptations and Online Modifications	Quantitative	Cross-sectional	Medical Students	104	COVID-19
74	Shin, T	2020	USA	Educational Adaptations and Online Modifications	Quantitative	Interventional Design	Medical Students	16	COVID-19
75	Sindiani, A	2020	Jordan	Educational Adaptations and Online Modifications	Quantitative	Cross-sectional	Medical Students	2212	COVID-19
76	Singh, K.	2020	India	Educational Adaptations and Online Modifications	Quantitative	Cross-sectional	Medical Students	208	COVID-19
134	Soltan, E	2020	Egypt	Student Knowledge and Attitudes	Quantitative	Cross-sectional	Medical Students	283	COVID-19
159	Stanislawski, E	2020	USA	Mental Wellness	Quantitative	Cross-sectional	Medical Students	200	COVID-19
77	Steehler, A	2020	USA	Educational Adaptations and Online Modifications	Quantitative	Cross-sectional	Medical Students	12	COVID-19
135	Taghrir, M	2020	Iran	Student Knowledge and Attitudes	Quantitative	Cross-sectional	Medical Students	240	COVID-19
136	Torun, F	2020	Turkey	Mental Wellness + Student Knowledge and Attitudes	Quantitative	Cross-sectional	Medical Students	275	COVID-19
137	Tran, B	2020	Vietnam	Student Knowledge and Attitudes	Quantitative	Cross-sectional	Medical Students, Medical Professionals, Community Workers	487	COVID-19
160	Vahedian, A	2020	Iran	Mental Wellness	Quantitative	Cross-sectional	Medical Students, Medical Staff, Patients, Public	207	COVID-19
78	Walton, R	2020	USA	Educational Adaptations and Online Modifications	Quantitative	Cross-sectional	Medical Students	50	COVID-19
79	Wang, C	2020	China	Educational Adaptations and Online Modifications	Quantitative	Cross-sectional	Medical Students	118,080	COVID-19
161	Wang, Y	2020	China	Mental Wellness	Quantitative	Cross-sectional	Undergradute Students, Postgraduate Students, and Residents in Medicine, Medical Technology, and Nursing	940	COVID-19
80	Wilcha, R	2020	United Kingdom	Educational Adaptations and Online Modifications	Qualitative	Systematic Review	Medical Students	Not Applicable	COVID-19
81	Williams, C	2020	USA	Educational Adaptations and Online Modifications	Mixed methods	Interventional Design; Content Analysis	Medical Students	10	COVID-19
162	Wong, J	2004	Hong Kong	Mental Wellness	Quantitative	Cross-sectional	Health Students, Non-Health Students	159	SARS
138	Wong, T	2007	Hong Kong	Student Knowledge and Attitudes	Quantitative	Cross-sectional	Medical Students	190	SARS
163	Wong, T	2005	Hong Kong	Mental Wellness	Quantitative	Cross-sectional	Health Students, Non-Health Students	169	SARS
164	Wu, S	2020	China	Mental Wellness	Quantitative	Cross-sectional	Medical Students, Medical Staff	201	COVID-19
139	Xiao, H	2020	China	Student Knowledge and Attitudes + Mental Wellness	Quantitative	Cross-sectional	Medical Students	620	COVID-19
165	Xie, L	2020	China	Mental Wellness	Quantitative	Cross-sectional	Medical Students, Non-Medical Students	805	COVID-19
82	Yang, T	2020	USA	Educational Adaptations and Online Modifications	Quantitative	Cross-sectional	Medical Students, Medical Staff, Faculty	48	COVID-19
166	Ye, W	2020	China	Mental Wellness	Quantitative	Cross-sectional	Medical Students, Non-Medical Students	2498	COVID-19
140	Yu, N	2020	China	Student Knowledge and Attitudes + Student Involvement and Telehealth	Quantitative	Cross-sectional	Medical Students	552	COVID-19
83	Zhang, Q	2020	China	Educational Adaptations and Online Modifications	Quantitative	Interventional Design	Medical Students	48	COVID-19

Seven major themes, with multiple subthemes, emerged from the thematic analysis among the included studies:

- Educational adaptations and online modifications (subthemes: preclinical and clinical adaptations, instructional methods and technology tools used for online learning, and successes, challenges, and student satisfaction with online learning);
- Knowledge and attitudes of students (subthemes: public health and preventive measures, student knowledge, resources of information, perceived fear and anxiety, effect on career choices, and comparisons between medical students and non-medical students);
- Mental wellness of students (subthemes: general mental wellness and stressors in response to the pandemic as well as stressors related to pivoting to online learning);
- Student involvement and use of telehealth (subthemes: reasons to or not to participate in activities during the respective pandemic(s), the roles and engagements students participated in, and student takeaways from being involved);
- Student vaccination (subthemes: vaccine uptake by medical students; reasons for and against vaccination; and influenza knowledge and preventive behaviors);
- Physical wellness of students; and
- Stigma.

The remainder of the results section provides a more detailed overview of each theme and subtheme.

### Theme 1: educational adaptations and online modifications

The most prevalent theme that emerged in this review was related to educational adaptations and online modifications to medical student education during a pandemic, with 69 studies reporting on school response, student attitudes towards and satisfaction of educational activities, curricular adaptations, instructional methods and technology, and general challenges and successes in medical education during a pandemic [3,16–83]. All studies focused on the COVID-19 pandemic, with the exception of one study that focused on COVID-19, SARS, and MERS [25] and all were published in 2020. Fifty-one studies were quantitative using cross-sectional survey [2,3,16,17,19–24,26–32,36–39,41–44,48,50–52,55–60,62,64–67,69,70,73–79,82,83]. Six studies were qualitative, [33,40,47,49,53,68] six were mixed methods, [18,54,61,63,71,81] and six were systematic reviews [25,34,35,45,46,80]. All studied either medical students alone (n = 52) [3,17,18,20–32,35–37,40,41,44,47,49–58,60,62–65,67,69–71,73–81,83] or compared medical students with other health-care populations including nursing, dental, pharmacy, and health science students, residents, fellows, and faculty (n = 17) [2,16,19,33,34,38,39,42,43,45,46,48,59,61,66,68,82].

Three common subthemes emerged: (1) preclinical and clinical adaptations, (2) instructional methods and technology tools used for online learning, and (3) successes, challenges, and student satisfaction with online learning.

#### Preclinical and clinical adaptations

Nearly half of the studies (n = 32) reported on curricular adaptations with five directly impacting preclinical education [40,53,56,57,69], 25 directly impacting clinical education [2,3,18,19,22,28–30,33,36,37,41,48,52,54,55,58,63,68,70,74,77,78,81,82], and two impacting non-required activities meant to supplement the curriculum [24,60]. Preclinical curricula that shifted to online formats included anatomy flipped classrooms [69], general classroom sessions (lectures and assessments) [57], healthcare improvement and safety curricula [53], telemedicine clinical skills with virtual OSCEs [56], and prerecorded general practitioner visits with patients [40]. Common adaptations in the clinical years included incorporating telehealth, [2,18,30,33,82] live streaming [48], online cases [37,74], online standardized patient encounters and OSCEs to assess communication skills [63], online interprofessional education activities [68], virtual rotations [28,81], and updated exam formats (online open book exams vs. in-person closed book exams) [3]. Adaptations in the clinical years impacted several disciplines including radiation oncology [2], radiology [19,22,36,41], urology [55,81], pathology [70], laboratory medicine [54], geriatrics [58], surgery [3,29,37,74], pediatrics [52,82], and otolaryngology [77]. Health policy curricula was also impacted [78]. Two studies focused less on specific required curricula and more on co-curricular activities that were implemented as a result of adaptations needing to be made to in-person activities and included virtual conversations to allow students to interact with physicians [24] and an internship bootcamp for M4s to feel better prepared and more confident for residency [60].

#### Instructional methods/technology tools

The shift to online learning was accompanied by experimenting with an assortment of online applications and management systems for asynchronous and synchronous learning. There were 26 total studies describing the various electronic instructional methods and technological adaptations used [16,18,20,23,25,27,30,33–35,39,42,44,47,48,51–53,57,58,61,62,65,69,73,76]. With regard to management systems, two studies described using Moodle [20,51] and one study each described utilizing Google Suite (Google, LLC, US) [76] and Microsoft Box (Microsoft Corp., US) [53] to help facilitate clinical experiences. Zoom (Zoom Video Communications, Inc., US) meetings were used for a variety of purposes, including online classes [20,25,27,30,34,41,48,69], online conferences [24], and live streaming surgeries [48]. Other live video conference tools used were Adobe Connect (Adobe, Inc., US) [51], Skype (Microsoft Corp., US) [20,69], Google Meet (Google, LLC, US) [65,76], Impartus (Impartus, India) [65], Microsoft Teams (Microsoft Corp., US) and AccuRx (AccuRx, Great Britain) [33]. One study found that video lectures were the preferred option by students compared to recorded lectures in Microsoft PowerPoint form [57]. In order to supplement clinical experiences, four studies described using Aquifer (Aquifer, Inc., US) cases [30,52,58,73]. To aid communication, utilizing applications like WhatsApp Messenger (Meta Platforms, Inc., US)[20], Facebook (Meta Platforms, Inc., US) groups [20], and Slack (Slack Technologies, LLC, US) [61] were also reported. YouTube (Google, LLC, US) was used in four studies for students to access recorded tutorials, class, and educational materials [20,39,65,69]. Furthermore, online question banks were used to supplement online learning [30,39,47]. In order to access online resources, students used several devices such as laptops and computers [23,25,73], as well as mobile devices [16,25,62].

#### Successes, challenges, and student satisfaction with online learning

Nearly all studies in this sub-theme reported some success and/or challenge to transitioning these educational adaptations to the virtual environment. The most commonly reported positive outcome of moving to an online teaching and learning environment described by eight studies was the convenience and flexibility offered by this model including no traveling, saving time, and being in the comfort of their own homes [30,33,35,39,46,49,70,73]. This was followed by five studies reporting an increase in the number of study resources and materials developed and available [43,45,65,70,80] and five studies reporting a positive impact on self-directed learning and productivity [23,35,43,45,80]. Institutions and faculty reported that the pandemic afforded the opportunity to try new methods of teaching and assessment [35,45,80] and both students and faculty believed most technology was easy to use and adopt [23,46]. Some studies also reported unique successes including increased time to work on research projects [45] and to spend with family [49] as well as the reduced costs of working and learning at home [39].

One area that was identified as a success by some studies and a challenge by others was the virtual engagement between students, teachers, and peers. Two studies reported improved interactions [20,46] while four studies reported poor communication in the virtual teaching environment [20,39,49,75]. However, the most challenging aspect of adapting to the online environment was technology-related issues such as poor internet connectivity, power outages, and learning curve of technology tools reported in 19 studies [20,23,27,33,35,38,39,42,45,46,49,59,65,66,69–71,75,80]. This was followed by seven studies each reporting the negative impact of pivoting to online learning on clinical and practical experiences [23,33,46,49,59,65,80] and the increased stress and anxiety of the pandemic on students [17,23,39,42,46,66,75]. Other reported challenges included: distractions/disturbances at home [33,38,39,49], lack of motivation [35,38,80], online fatigue [38,49], low quality of teaching [20,49], and financial strain of buying technology [23].

Multiple studies explored student’s perceptions regarding the role of E-learning in comparison to traditional classrooms. Overall, there was no consensus on whether online learning was more preferable than traditional classroom. Three studies stated that a majority of students found online classrooms to be better than in-person [23,26,71]. Seven studies stated that face-to-face classroom was preferred over online [16,50,57,62,67,76,83]. Several studies found that some combination of online and in-person is preferable by students for the future [20,66,71,83]. One study explored the various factors affecting students’ desire to return to traditional teaching methods [31]. Overall, satisfaction amongst medical students regarding the transition to online learning varied greatly with several studies reporting high satisfaction while others reported low satisfaction [20,21,32,51,71]. Various factors affected the level of satisfaction such as technical difficulties [71], and students’ previous experience and familiarity with distance learning [20,79]. Two additional studies focused on student satisfaction related to the overall school response to the pandemic [26,64]. Both studies reported mixed levels of student satisfaction and revealed three primary areas of dissatisfaction: 1) lack of transparent school communication; 2) lack of training or information about PPE and COVID-19 in general; and 3) anxiety about their safety and risk of infection.

### Theme 2: student knowledge and attitudes

The second largest represented theme in this review was the exploration of knowledge and attitudes of medical students during their respective pandemic. A total of 59 studies were included in this theme published between 2002 and 2020 [26,64,84–140]. Multiple pandemics were the focus of this theme, with the majority focused on COVID-19 (n = 35) [26,64,84,85,89,91,92,94,95,97,98,102,104,109,111,112,114,116,118,119,121–125,131–137,139–141]; H1N1 (n = 8) [106,107,110,117,120,126,128,129]; Ebola (n = 6) [86,93,105,108,115,127]; MERS (n = 5) [87,88,90,100,101]; SARS (n = 3) [96,113,138]; and Zika (n = 2) [103,130]. Almost all study types were cross-sectional and study methods were quantitative except one study which was a qualitative systematic review [91] and one a mixed-method study[117]. Thirty-three studies focused solely on medical students [26,64,84,87,88,91,93–96,100,106,107,109–116,120,122,123,127,131,133–136,138–140], while 26 studies explored knowledge and attitudes of medical students and other students and professionals such as attending physicians, nursing students, and dental students [85,86,89,90,92,97,98,101–105,108,117–119,121,124–126,128–130,132,137,141].

Common sub-themes that arose in this theme included public health and preventive measures, student knowledge, resources of information, perceived fear and anxiety, effect on career choices, and comparisons between medical students and non-medical students.

#### Public health and preventive measures

A total of 12 studies discussed preventive measures [84,88,89,95,102,104,105,115,117,121,129,138]. The most frequently identified types of the preventive measures were handwashing (n = 9) [84,89,95,102,104,115,117,121,138] and wearing masks (n = 8) [88,89,95,102,105,117,121,129], followed by isolation (n = 4) [88,102,117,129].

The nine handwashing studies were related to COVID-19 (n = 6), Ebola (n = 1), SARS (n = 1), and H1N1 (n = 1) [84,89,95,102,104,115,117,121,138]. In all but one of the nine studies, handwashing was utilized or identified as an effective preventive measure by a vast majority of medical students [84,89,102,104,115,117,121,138]. However, one study found that only about half of the respondents identified handwashing as the basic step for prevention of Ebola [115]. Three studies indicated increased use of hand-hygiene practices following the SARS, MERS, and H1N1 pandemic respectively [88,117,138]; however, there were discrepancies between knowledge and practice [138]. In addition, two studies mentioned the use of hand sanitizer [89,117]. One study regarding the COVID-19 pandemic noted that almost all of students wash their hands and use alcoholic rub [89]. During the H1N1 pandemic, another study demonstrated an increased use of hand sanitizer as the most frequently increased behavior in patient care activities whereas handwashing was the most commonly increased behavior at home [117]. In one study, almost all respondents were able to correctly identify that the duration of handwashing is at least 20 seconds as recommended by the Centers for Disease Control and Prevention [121].

Of the seven studies in which masks were known to be or actually used as a preventive measure, four were related COVID-19 [89,95,102,121], one from Ebola [105], and two from H1N1 [117,129]. Four of these seven mentioned N95 masks usage [89,95,117,121] whereas the remainder identified only general mask usage. One study regarding the H1N1 pandemic indicated that N95 masks were used less frequently than general surgical facemasks, while also demonstrating the smallest uptake of surgical facemasks [117]. The remaining six studies indicated that the majority of students were knowledgeable regarding the use and benefits of facemasks [89,95,102,105,121,129]. Two studies indicated that almost all of the students practiced proper cough etiquette [89,117]. Three studies demonstrated a wide range of knowledge regarding the use of cap, goggles, disposal gown, face shields, aprons, and shoe covers as PPE [95,105,121] with one study finding that only half of the students had adequate knowledge of these measures [105].

Studies comparing medical student and resident knowledge of preventive measures reported mixed results. One study indicated that residents had more knowledge regarding PPE than medical students [124]. Similarly, another study demonstrated that preventive behaviors increased with increased educational attainment and age [104]. This is in contrast to another study which showed that medical students were more knowledgeable about PPE than residents [85].

One study showed that if there was an epidemic in the institution, most students would isolate in some form, but less than half of medical students would isolate themselves from school and around half would isolate from social events [129]. Another study indicated just over half agreed to isolate from social gatherings or large crowds, while also demonstrating that the most common reason for not staying home was ‘not wanting to miss class/work’, being reported equally by students in pre-clinical and clinical years [117]. Majority of residents and students have reported no to minimal effect on grades or work performance [117]. One study mentioned medical students avoiding contact with patients as a form of isolation [102].

Three studies discussed education on preventive measures [26,104,122]. The majority of the medical students from one study attended training classes on hygiene in epidemic prevention and disaster prevention [122]. Despite this, another study demonstrated that policies are needed in order to better prepare medical students to contribute to the COVID-19 pandemic and beyond [104]. One study demonstrated that medical students who had participated in community service activities showed increased participation in epidemic sanitation training compared with those who did not [122]. One study also showed that students from public institutions received more training [26].

#### Student knowledge

Over multiple pandemics, medical student knowledge regarding each disease has explored topics such as transmission, symptoms, mortality, and treatment. Specifically, 23 studies explored student knowledge regarding methods of transmission [87,90,92,95,98,100–102,105,107,110,114,115,119,120,128,130,132–134,136,137,141]; 22 reported student knowledge of symptoms of various pandemics [87,89,90,95,98,100,102,107,110,114,118,120,122,126,127,129,130,132–134,137,141]; 11 looked at knowledge of treatment [90,95,100,102,105,110,114,119,127,128,140]; and three studies examined student knowledge regarding mortality [87,101,139]. Multiple pandemics were represented including COVID-19 (n = 17) [90,92,95,98,102,114,118,119,122,132–134,136,137,139–141]; H1N1 (n = 4) [107,110,120,128]; MERS (n = 4) [87,90,100,101]; Ebola (n = 3) [105,115,127]; Zika (n = 1)[130].

Rates of knowledge regarding the pandemics varied between studies, but almost all studies found that the majority of students were knowledgeable regarding basics of transmission, symptoms, mortality, and treatment of their respective pandemics. With regard to transmission, most were knowledgeable about most modes of transmission but their understanding could improve regarding transmission via objects contaminated with the virus [120]. With regard to symptoms, only one study found that the majority of students did not know the symptoms of MERS [100]. Another study found that the majority of students were not aware of the mortality rate of MERS [101]. Two studies demonstrated the increased need for knowledge regarding treatment [90,100]. Overall knowledge of the various diseases was mostly sufficient with only two demonstrating poor knowledge [130,136]. One study demonstrated a correlation between related knowledge and practicing preventive behavior [134]. With varying levels of knowledge, seven studies recommended increasing educational and awareness programs to further advance student knowledge [87,95,100,103,105,110,140].

#### Resources of information

Seven studies discussed resources of information in learning and keeping up with emerging updates about H1N1,^110^ MERS [87,88], and COVID-19 [84,95,100,136]. The information source reported most frequently for COVID-19 and MERS was social media with the majority of information regarding H1N1 coming from television. Other information sources reported for COVID-19 include websites run by the government, institutional announcements for MERS and for H1N1; news articles were also cited as important sources for information.

#### Medical students vs. non-medical students

A total of 21 studies directly compared differences in knowledge and attitudes of medical students and other students and professionals [85,89,90,92,98,101–104,117–119,121,125,127–130,132,137,141]. Ten studies found that medical students had more knowledge regarding the respective pandemic being assessed than non-medical students [90,92,101,102,105,119,127,130,132,141]. However, two studies found no difference between medical students and non-medical students [89,129]. When medical students were compared to other professionals such as residents, interns, attendings, or lecturers, three studies found that medical students scored lower in knowledge and attitude levels [118,121,128]. Three studies found that other professionals were more likely to adopt more protective habits, such as the use of antiseptics and masks, compared to medical students, [98,104,117] while one study demonstrated that medical students and nurses were more knowledgeable about PPE than other health-care workers [85]. One study found that medical students had less knowledge regarding COVID-19 guidelines compared to medical professionals and community workers, due in part to medical professionals and community workers receiving information from their respective organizations directly [137].

#### Perceived fear and anxiety regarding infection

Pandemics have the potential to cause significant stress for individuals in general, and medical students are no exception. Nine studies focused on medical student stress and anxiety during multiple pandemics [26,64,93,95,96,120,123,124,136]. The majority of studies focused on students’ fear of becoming infected. Interestingly, four of the studies were able to demonstrate that along with a reported increase in knowledge, students’ levels of fear and anxiety were lowered [93,95,123,136]. Only one study showed the inverse, in that residents in this particular study were more fearful than medical students, although presumably possessing more knowledge and experience [124]. One study explored fear of providing mouth resuscitation Basic Life Support during the SARS pandemic and found that students were more reluctant to provide mouth resuscitation due to SARS [96]. In cases where gender was differentiated, women consistently showed higher levels of anxiety [123,124,136].

### Theme 3: mental wellness

Another theme that emerged in this review was related to mental wellness of medical students with 31 studies reporting on general mental wellness and mental wellness specifically related to pivoting to online learning [17,42,112,136,139,141–166]. While most studies (n = 27) focused on the COVID-19 pandemic, there were three studies that focused on SARS [148,162,163] and one study that focused on Ebola [149]. All 27 COVID-19 studies were published in 2020. The SARS studies were published in 2004 [148, 162] and 2007^163^ and represented two unique countries: China (n = 1) [148] and Hong Kong (n = 2) [162,163], while the Ebola study was published in 2017 from Sierra Leone [149]. Twenty-six COVID-19 studies were quantitative using cross-sectional [17,75,112,136,139,141–147,150,152–157,159–161,164–166] and one interventional design [158]. One study was a systematic review [151]. All of the SARS studies [148,162,163] and the Ebola study [149] were also quantitative using cross-sectional surveys.

Nineteen COVID-19 studies focused on medical students alone [17,112,136,139,141–143,146,147,150–159] while eight compared medical students with other student populations including nursing, dental, pharmacy, applied medical science and non-medical students (n = 5) [42,145,161,165,166], as well as residents, faculty, community members, and patients (n = 3) [144,160,164]. All three SARS studies included medical students and non-medical/non-health students [148,162,163] and the Ebola study included medical students and staff [149].

Two common subthemes emerged regarding mental wellness: general mental wellness and stressors in response to the pandemic as well as stressors related to pivoting to online learning.

#### General mental wellness and stressors in response to the pandemic

The majority of the studies in this theme measured medical student psychological status/mental well-being during a pandemic (n = 24) [136,139,141,142,144,145,147–149,151–156,158–166] Three studies assessed psychological status during SARS [148,162,163] and one during Ebola [149] with the remainder reporting during the COVID-19 pandemic. All 24 studies identified changes in medical student mental well-being through studying one or more psychological states: depression and anxiety were the most frequently measured with 11 studies assessing for depression [139,141,145,147,149,152,155,156,158,160,165] and 11 looking at anxiety [139,141,147,151,152,155,156,158,160,163,165]. This was followed by assessing psychological stress or distress levels in nine studies [136,147,153,154,158–162] and changes in sleep patterns in four studies [136,145,147,158]. Another seven studies did not identify specific disorders, but assessed general psychological wellness of medical students [142,144,145,148,154,164,166]. Three studies looked beyond measuring psychological state to gathering student coping and stress management strategies [142,144,159].

Some studies compared the mental well-being of medical students by gender and/or class year. Of those studying gender, six studies found that females demonstrated higher levels of depression, anxiety, and stress compared to males [139,147,153,155,156,159]; while one study found anxiety was higher in men [160]. In contrast, five additional studies found no statistically significant differences between women and men [141,145,152,160,162]. Of the three studies assessing differences by class year, two studies found no differences between class years [141,153] while one found that third-year medical students reported lower levels of stress than first-year students [159].

As all studies were cross-sectional surveys, there were several commonly reported instruments used to measure these various psychological factors. The Generalized Anxiety Disorder (GAD-7) was most reported (n = 7) [139,141,147,152,156,159,165] followed by the Patient Health Questionnaire-9 (PHQ-9) (n = 5) [141,147,152,159,165]. This was followed by versions of the Kessler Psychological Distress Scale (n = 4) [147,153,161,164] and the Perceived Stress Scale (n = 3) [136,162,166]. The Impact of Event Scale-Revised (IES-R) was used in three studies [154,161,165] followed by the Center for Epidemiology Studies for Depression Scale (CES-D) [154,156], Depression Anxiety Stress Scale (DASS-21) [158,160], and Symptom Checklist 90 (SCL-90) [148,149] being used as measures in two studies each.

#### Mental wellness/stressors related to pivoting to online learning

Nearly one-quarter of the studies focused on mental wellness and stressors (general stress, anxiety, depression, post-traumatic stress disorder, and burnout) related to pivoting to online learning during medical school and all were related to COVID-19 (n = 7) [17,42,112,143,146,150,157]. Most studies primarily focused on the stressors of online learning in general [17,112,143,146,150] though two studies focused on stressors and burnout specifically related to new lecture systems and assignments [157] as well as remote exams [42].

### Theme 4: student involvement and telehealth

With day-to-day activities of medical students being affected by their respective pandemic, medical students explored various outlets to further their development as future physicians and contribute to the community. Sixteen studies published between 2005 and 2020 examined how medical students stayed involved during their respective pandemic [18,33,73,84,95,140,167–176]. Pandemics studied included: COVID-19 (n = 14) [18,33,73,84,95,140,167–173,176], Ebola (n = 1) [175], and one study researching multiple pandemics: SARS, H1N1, MERS, Ebola, and COVID-19 [174]. Study methods utilized include quantitative (n = 11) [73,84,95,140,167–171,175,176], qualitative (n = 3) [33,173,174], and mixed methods (n = 2) [18,172]. Most study types were cross-sectional with a phenomenological study [173], a systematic review [174], an interventional design [18], and an interview-based design [33]. Among the 16 studies common themes included reasons to or not to participate in activities during the respective pandemic(s), the roles and engagements students participated in, and student takeaways from being involved.

#### Reasons to or not to get involved

The number of medical students involved during the pandemic varied, but four studies found that the majority of students were willing to or had volunteered to aid in the pandemic response [95,140,168,171]. Reasons medical students got involved included a sense of duty to society, caring for others, interest in medical activity, social commitment, learning opportunities, experience, participation in a historic event, and pride in contributing [140,168,171,176]. Reasons for not participating included lack of time due to studying or part-time jobs, concerns regarding PPE, risk of infection, possible increased burden to the hospital, and risk of infecting others [95,168,171,176]. One study found that about half of final year medical students were willing to join the workforce [167].

#### Roles medical students played

One of the most prominent outlets in which medical students engaged was through telehealth with seven studies examining such services [18,33,73,169–172]. During telehealth sessions, students helped screen, triage, answer questions, assess needs of underserved populations, and educate populations including those from rural and lower socioeconomic statuses [18,33,73,169–172]. Whether students preferred an indirect or direct role in the treatment of patients was uncertain with one study demonstrating that a majority preferred indirect [140] and another demonstrating that students were concerned about only performing indirect tasks [95]. Two studies identified that students participated in outreach programs to increase knowledge of Ebola [175] and SARS [174]. One study found that about half of final year students were willing to join the workforce [167]. Other student roles included volunteering in hospitals, data administration, intensive care units, emergency departments, ambulance services, and general practice [33].

#### Benefits of involvement

Participation in telehealth provided several benefits including improving student communication skills, increasing clinical exposure, gaining broad awareness of medicine and social determinants of health, as well as improved comfort in clinical responsibilities such as answering questions about COVID-19, conducting audio physical exams, triaging, screening patients, and addressing financial burdens to care [18,33,169,170,172]. One study found that through a community outreach program during Ebola, students and community members were more motivated to combat Ebola [175]. In one study, the majority of students involved in the program agreed that incorporating telehealth into the internal medicine clerkship would improve their experience [33]. Ultimately, medical students were able to find ways to remain engaged across various pandemics which have the potential to remain beneficial engagements in a post-pandemic world.

### Theme 5: student vaccination

Another theme that emerged in this review related to medical student vaccination with 12 studies reporting on vaccination uptake, knowledge, and behaviors in medical students during the 2009 H1N1 pandemic [117,120,126,129,177–184]. Studies were published between 2010 and 2018 with ten being quantitative in design using cross-sectional surveys (n = 9) and a prospective cohort design [184]. One study was a mixed method using both a survey and interviews [183]. All studied either medical students alone (n = 5) [120,178,181–183] or compared medical students with other health populations (n = 7) including health-care professionals [177], dental students [180], nursing students [179], nursing & midwifery students [126,129], university students [184], and medical residents [117].

Three common themes emerged among the 12 studies: vaccine uptake by medical students; reasons for and against vaccination; and influenza knowledge and preventive behaviors.

#### Vaccine uptake

All 12 studies reported on medical students’ H1N1 vaccine uptake or their intention to get vaccinated against the H1N1/swine flu. Uptake varied dramatically from 1.7%[180] to 93.2%[179] with only 4 studies reporting a 50% or greater vaccination rate in medical students in Brazil, the US, and Sweden [117,178,179,183]. In studies that compared medical students to other groups, medical students tended to have higher vaccination rates than other health students [126,179], health-care professionals [181], and the general public [182]. Only one study found a higher intention to vaccinate in nursing students compared to medical students, but did not collect actual uptake rates [129].

#### Reasons for and against getting the vaccine

Most studies also reported on medical student reasons for and against getting the vaccine (n = 8) [117,120,177–179,181–183]. The most commonly cited reason for getting vaccinated was protecting oneself, family, friends, and/or patients [120,179,181–183]. Reasons for not getting the vaccine varied widely with the top 4 reasons being: not perceiving themselves at risk of contracting it or having severe illness [117,177–179,181–183]; fear of adverse effects [117,120,177,178,181,182]; doubting vaccine effectiveness [120,177–179,182,183]; and inconvenience, such as access and time [120,177,178,181,183].

#### Knowledge of influenza, vaccines, and preventive behaviors

In addition to vaccine uptake, studies analyzed medical student knowledge and their behaviors regarding the H1N1 pandemic and the vaccine. Only one study reported high levels of influenza and vaccination knowledge in medical students [179]. Some studies found mixed results in medical student knowledge of disease transmission, symptomatology, vaccine safety and effectiveness, and preventive measures with many students holding common misconceptions [117,120,178,182]. However, four reported that knowledge and preventive practices improved as students progressed into their clinical years [126,181–183]. Across studies, many reported students could appropriately identify major preventive measures including increased handwashing, wearing of face masks, social distancing, cough etiquette, and isolation if sick, but reported adherence to these practices varied widely [117,120,129,180].

The primary lesson reported from this set of H1N1 pandemic literature was a call for increasing the training and education of medical students in influenza, its prevention, flu vaccine effectiveness and safety, and addressing misconceptions surrounding flu vaccine [126,177,178,180,182–184]. The study with the highest reported vaccination coverage of 93.5% credits their curriculum and interprofessional education with medical student vaccine adherence and high knowledge levels [179].

### Theme 6: physical wellness

Three studies explored physical wellness of medical students during the COVID-19 pandemic through the use of cross-sectional surveys [185–187]. In one study, it was found that the majority of students reported unchanged or more physical activity during the pandemic than before [185]. Eating habits were also explored and found that about half reported better eating behavior than before, and that healthy diet literacy was correlated with food choices during the pandemic [185]. Two studies explored sleeping habits of students and found that almost all of students reported adequate sleep at night during the pandemic and the majority of students stated an increase in time asleep [186,187]. An increase in screen time during the pandemic was reported by the majority of students [187].

### Theme 7: stigma

A single study examined the stigma associated with race during the COVID-19 pandemic through a cross-sectional survey [188]. Specifically, the study found that 61.2% of Asian students experienced prejudice in Poland, with public transportation being where the most (47.1%) prejudice was encountered. Additionally, 21.2% of Asian medical students reported facing prejudice by patients and being asked if they carried the virus themselves. Nearly one-quarter (24.7%) of Asian medical students reported facing prejudice at the university where they study [188].

## Discussion

The seven key themes identified from the included studies originated from numerous countries around the world and were primarily focused on COVID-19, but also represented the other major pandemics and epidemics since 2000. Although the impact of COVID-19 was far greater than other pandemics, there are still important parallels that can be drawn. Within each theme lie potential takeaways, as well as avenues for further exploration that can be utilized to improve medical education.

### Theme 1: online education/educational adaptations

Online education and educational adaptations were distinctly divided between pre-clinical and clinical education, with clinical adaptations receiving more attention in the literature. While pre-clinical adaptations were mostly related to classroom learning (lectures, flipped classroom) [40,53,56,57,69], clinical adaptations focused on patient interaction (telehealth, standardized patient encounters) [2,3,18,19,22,28–30,33,36,37,41,48,52,54,55,58,63,68,70,74,77,78,81,82] and interprofessional opportunities [68]. Additionally, there was a focus on online tools and platforms used to make these adaptations [16,18,20,23,25,27,30,33–35,39,42,44,47,48,51–53,57,58,61,62,65,69,73,76]. A range of successes and challenges were also highlighted. Most of the online education/adaptation studies were related to COVID-19 and not SARS, MERS, Ebola, or H1N1. This may be a result of the more pervasive impact that COVID-19 has had globally than the other pandemics that were investigated, as well as the availability and accessibility of online tools during COVID-19. It is evident from the literature that some online educational adaptations provide greater learning opportunities and flexibility for learners and will likely remain as part of the medical education landscape post-COVID-19. In addition, further studies will be helpful regarding communicating effectively with students in the virtual platform, for delivery of curricular content, and information regarding the pandemic.

### Theme 2: student knowledge and attitudes

Studies on this theme primarily focused on public health and preventive measures [84,88,89,95,102,104,105,115,117,121,129,138], as well as student knowledge and attitudes regarding the respective pandemic [87,89,90,92,95,98,100–102,105,107,110,114,115,118–120,122,126–130,132–134,136,137,141]. Across pandemics, handwashing and mask wearing were the primary foci, with residents being more knowledgeable than medical students regarding preventive measures. Studies related to student knowledge and pandemics focused on four main themes: transmission, symptoms, mortality, and treatment. In general, students had sufficient knowledge regarding transmission and symptoms, yet were not fully aware of the magnitude of mortality and were also not sufficiently aware of possible treatment options. However, medical students tended to display a better general understanding regarding their respective pandemic than other students [90,92,101,102,105,119,127,130,132,141], while having less of an understanding than other health professionals [118,121,128]. Studies were able to demonstrate that along with a reported increase in knowledge, students’ levels of fear and anxiety were lowered, suggesting the more knowledge one possesses, the less anxiety one feels.

Though classes on preventive measures and disease prevention were reported [26,104,122], many studies recommended increased training to address gaps in medical student knowledge and alleviate fears [87,95,100,103,105,110,140]. This supports the need for additional training opportunities for medical students in the standard curriculum post-pandemic. In addition, with rapidly emerging health information, increased emphasis should be placed on recognizing proper sources of information within the curriculum.

### Theme 3: mental wellness

Mental wellness was a focus of numerous studies related COVID-19^17^ [75,112,136,139,141–147,150–161,164–166], with a few related to SARS or Ebola [148,149,162,163]. While these studies focused on different aspects of mental health, including general stressors related to the pandemic in general [136,139,141,142,144,145,147–149,151–156,158–166] and to pivoting to online learning specifically [17,42,112,143,146,150,157], the focus of these studies was descriptive and on self-report of current circumstances and did not thoroughly explore strategies for successfully addressing these stressors in the medical student population. Future studies focusing on how to help students cope and teach them stress management strategies should be pursued. A few studies explored student coping strategies [142,144,159], which could be further explored to expand programs to reduce anxiety, depression, and burnout as a result of future pandemics.

### Theme 4: telehealth and student involvement

Student involvement and telehealth were explored in many studies with COVID-19 being the main focus of the majority of studies [18,33,73,84,95,140,167–173,176]. One of the most common ways students remained involved in patient care during the pandemic was through the use of telehealth services [18,33,73,169–172]. Students also participated in various other opportunities such as volunteering in hospitals [33]; however, telehealth was the most represented involvement of medical students, likely due to the versatility and ability to remain socially distanced. The use of telehealth allowed students to supplement their learning as well as serve the community. Students remained involved due to a variety of reasons including a desire to further learn and give back to the community, while some did not due to fears of lack of PPE and infection in face-to-face activities. Although limited, two studies regarding Ebola [175] and SARS, H1N1, MERS, Eboals, and COVID-19^174^ showed that students were involved in community educational programs in order to increase general public knowledge regarding the respective diseases which was also a significant role that medical students were able to fill during the COVID-19 pandemic. With the increased significance of telehealth encounters during the pandemic, telehealth serves as an opportunity for students to remain involved in community outreach and further develop clinical skills.

### Theme 5: student vaccination

Only studies related to the H1N1 vaccine were represented in this review due to the final search end date of December 2020. At which time, COVID-19 vaccinations were just being made available to health-care workers in the United States and globally even later. However, both H1N1^117^ [120,126,129,177–184], and COVID-19^87^ [90,95,103,105,110,120,140], studies found students lacked a comprehensive knowledge about the current disease of the time and points to the need for increased education in infectious disease prevention and treatment. A major difference between H1N1 and COVID-19 observationally is vaccination uptake. For H1N1, uptake varied dramatically for medical students, but emerging COVID-19 literature points to higher vaccination rates [189]. This difference could be attributed to the pervasiveness of COVID-19, vaccine mandates for health-care workers, and obligation to protect patients and family. However, literature published after completion of this scoping review still demonstrate medical student hesitancy toward the COVID-19 vaccine over concerns such as potential side effects, safety, and efficacy of the newly developed vaccine [190–193]. In addition, decreased student knowledge regarding vaccines and the COVID-19 vaccine itself was found to be correlated with vaccine hesitancy [190,191]. Future studies could explore this further and directly compare student vaccination knowledge, attitudes, and rates between COVID-19 and prior pandemics.

### Themes 6 & 7: physical wellness and stigma

Other themes that were explored in our study were physical wellness [185–187] and stigma [188]. Physical wellness was explored with reports of adequate and increased amounts of sleep, increased screen time, and unchanged physical activity, but further exploration is needed to evaluate the impact COVID-19 has had on physical wellness. Only one study examined the stigma that Asian medical students faced during the COVID-19 pandemic in Poland [188]. The study found that the majority of students did face prejudice related to the pandemic, but there is potential for further research of the role stigma has played on Asian medical students as research related to COVID-19 has continued to increase after our search end date of December 2020.

### Limitations

This scoping review has its limitations, primarily that the search only included studies published through 1 December 2020. An abundance of COVID-19 research was published in a relatively short period of time from March 2020 – December 2020 and it is likely that this pace continued or increased during 2021 and may answer some of the outstanding questions highlighted in the discussion, particularly related to student vaccination uptake, knowledge, and attitudes. In addition, as English-language studies were only included, some language bias may be introduced and not all relevant literature may have been included. Finally, as the scoping review methodology excludes quality assessment of included studies, the quality of the articles included in this review is mixed. Observationally, many studies, particularly published on COVID-19, were not well designed or reported; this could impact the level of bias present in the review. It showcases the opportunity for future studies to employ more rigorous methods in assessing the impact of pandemics on medical students.

## Conclusion

This scoping review explored how pandemics and epidemics since 2000 including SARS, H1N1, MERS, Ebola, Zika, and COVID-19 impacted medical students, both from an educational standpoint and personally. Pandemics prior to COVID-19 affected medical students at a regional level in many of the same ways that COVID-19 affected medical students worldwide. This included interruptions and adaptations to their education; their attitudes and knowledge related to the current pandemic, preventive measures, and vaccines; and new roles in aiding pandemic relief. Despite these similarities and potential lessons of previous pandemics, the regional nature of SARS, MERS, Ebola, and Zika and the less severe and pervasive H1N1 pandemic did not prompt medical schools to plan or prepare for COVID-19. Unique to the COVID-19 pandemic were technology opportunities and the mental health challenges associated with long-term isolation and disruptions. In particular, advancements in technology allowed medical schools to quickly adapt and modify preclinical and clinical experiences to the online learning environment; something that may not have been possible at the time of the other pandemics. It also highlights the ongoing need to prepare students for emergency situations and develop good stress management and coping strategies. Both of these areas present opportunities for medical schools to integrate more content into the curriculum. As well-stated by two systematic reviews published early in the pandemic: ‘This unprecedented circumstance will change the way in which we deliver medical teaching’, [45] and ‘though not all will be different, this turning point has increased faith in technology sparking a change in behaviour away from traditional approaches’.[46]

## Data Availability

The complete data extraction table associated with this article is available from the corresponding author. Victoria C. Lucia, lucia@oakland.edu, Oakland University William Beaumont School of Medicine, 586 Pioneer Drive Rochester, MI 48309.

## Appendix A. Search Strategies in All Databases

**Table:**

Database	Search strategy	Filter(s) applied
**Cochrane Library**	(medical education OR ‘undergraduate medical education’ OR medical student* OR medical school*) AND (SARS OR ‘severe acute respiratory syndrome’ OR ebola* OR H1N1 OR ‘influenza A’ OR MERS OR ‘Middle Eastern Respiratory Syndrome’ OR COVID* OR coronavirus* OR pandemic* OR epidemic* OR outbreak*)	None
**Dissertations & Theses** (ProQuest)	((title(‘medical education’ OR ‘undergraduate medical education’ OR ‘medical student’ OR ‘medical students’ OR ‘medical school’ OR ‘medical schools’) OR ab(‘medical education’ OR ‘undergraduate medical education’ OR ‘medical student’ OR ‘medical students’ OR ‘medical school’ OR ‘medical schools’)) AND noft(SARS OR ‘severe acute respiratory syndrome’ OR ebola* OR H1N1 OR ‘influenza A’ OR MERS OR ‘Middle Eastern Respiratory Syndrome’ OR COVID* OR coronavirus* OR pandemic* OR epidemic* OR outbreak*))	- English language - Publication years 1/1/2000-12/1/2020
**EMBase**	(‘medical education’:ab,ti OR ‘undergraduate medical education’:ab,ti OR ‘medical student’:ab,ti OR ‘medical students’:ab,ti OR ‘medical school’:ab,ti OR ‘medical schools’:ab,ti OR ‘medical students’/exp OR ‘medical school’/exp OR ‘undergraduate medical education’/exp) AND (SARS OR ‘severe acute respiratory syndrome’ OR ebola* OR H1N1 OR ‘influenza A’ OR MERS OR ‘Middle Eastern Respiratory Syndrome’ OR COVID* OR coronavirus* OR pandemic* OR epidemic* OR outbreak* OR ‘Coronavirus infection’/exp OR ‘covid 19’/exp OR ‘2009 H1N1 influenza’/exp OR ‘epidemic’/exp OR ‘pandemic’/exp)	- English language - Publication years 1/1/2000-12/1/2020
**ERIC** (ProQuest)	(medical education OR ‘undergraduate medical education’ OR medical student* OR medical school* OR SUBJECT.EXACT(‘Medical Students’) OR SUBJECT.EXACT(‘Medical Schools’) OR SUBJECT.EXACT(‘Medical Education’)) AND (SARS OR ‘severe acute respiratory syndrome’ OR ebola* OR H1N1 OR ‘influenza A’ OR MERS OR ‘Middle Eastern Respiratory Syndrome’ OR COVID* OR coronavirus* OR pandemic* OR epidemic* OR outbreak*)	- English language - Publication years 1/1/2000-12/1/2020
**PubMed**	(‘Education, Medical, Undergraduate’[Mesh] OR ‘Schools, Medical’[Mesh] OR ‘Students, Medical’[Mesh] OR medical education[tiab] OR medical student*[tiab] OR medical school*[tiab]) AND (SARS OR severe acute respiratory syndrome OR ‘SARS Virus’[Mesh] OR ebola* OR ‘Ebolavirus’[Mesh] OR ‘Hemorrhagic Fever, Ebola’[Mesh] OR H1N1 OR influenza A OR ‘Influenza A Virus, H1N1 Subtype’[Mesh] OR ‘H1N1 virus hemagglutinin’ [Supplementary Concept] OR MERS OR Middle Eastern Respiratory Syndrome OR ‘Middle East Respiratory Syndrome Coronavirus’[Mesh] OR COVID* OR coronavirus* OR ‘Coronavirus’[Mesh] OR ‘Coronavirus Infections’[Mesh] OR ‘COVID-19’ [Supplementary Concept] OR pandemic* OR epidemic* OR outbreak* OR ‘Disease Outbreaks’[Mesh])	- English language - Publication years 1/1/2000-12/1/2020
**Google Scholar**	(‘medical education’ OR ‘medical student’ OR ‘medical school’) AND (SARS OR ‘severe acute respiratory syndrome’ OR ebola OR H1N1 OR ‘influenza A’ OR MERS OR ‘Middle Eastern Respiratory Syndrome’ OR COVID OR coronavirus OR pandemic OR epidemic OR outbreak)	- Publication years 1/1/2000-12/1/2020
**Northern Lights Conference Abstracts** (OVID)	(medical education OR medical student OR medical school) AND (SARS OR severe acute respiratory syndrome OR ebola OR H1N1 OR influenza A OR MERS OR Middle Eastern Respiratory Syndrome OR COVID OR coronavirus OR pandemic OR epidemic OR outbreak).ti OR (medical education OR medical student OR medical school) AND (SARS OR severe acute respiratory syndrome OR ebola OR H1N1 OR influenza A OR MERS OR Middle Eastern Respiratory Syndrome OR COVID OR coronavirus OR pandemic OR epidemic OR outbreak).ab	None
**Scopus**	TITLE-ABS((‘medical education’ OR ‘undergraduate medical education’ OR ‘medical student’ OR ‘medical students’ OR ‘medical school’ OR ‘medical schools’) AND (SARS OR ‘severe acute respiratory syndrome’ OR ebola* OR H1N1 OR ‘influenza A’ OR MERS OR ‘Middle Eastern Respiratory Syndrome’ OR COVID* OR coronavirus* OR pandemic* OR epidemic* OR outbreak*))	- English language - Publication years 1/1/2000-12/1/2020
**Web of Science**	(‘medical education’ OR ‘undergraduate medical education’ OR medical student* OR medical school*) AND (SARS OR ‘severe acute respiratory syndrome’ OR ebola* OR H1N1 OR ‘influenza A’ OR MERS OR ‘Middle Eastern Respiratory Syndrome’ OR COVID* OR coronavirus* OR pandemic* OR epidemic* OR outbreak*)	- English language - Publication years 1/1/2000-12/1/2020

## Appendix B. Data Extraction Form

**Table:**

**Publication data**	
First author	Last Name, First Initial
Publication date	Year
Journal name	
Location of study	Country & State if US
**Purpose**	
Aims/Research question(s)	Description of study aims and/or research question
**Methods**	
Method type	Quantitative, qualitative, mixed method
Study design	Identify study design (systematic review, cross-sectional survey, focus groups, interviews, etc) and describe interventions
Statistical analysis method(s)	Identify statistical methods used (t-tests, Chi-square, Pearson, etc)
Entire study population *(if applicable – more than just medical students are included)*	List all populations included in the study (ie: physicians, nurses, medical students, health students, etc) or N/A
Medical student population	Description of medical student population (all years; M1, M2, M3, M4 (M5, M6 if international); preclinical, clinical) or not defined)
Sample size of total study population *(if applicable)*	# or N/A
Sample of size of medical students	#
Pandemic(s) discussed (select all that apply)	COVID-19, SARS, MERS, Zika, Ebola, H1N1/Swine Flu
**Results**	
General study outcomes/results	Description of all study outcomes/results or N/A if entire study was related to medical students
Key findings/lessons learned that specifically relate to our review	Description of key findings that related directly to our review of medical students & pandemics
